# The ethics of exclusion: why pregnant and lactating women must be front and centre of HIV research

**DOI:** 10.1002/jia2.25926

**Published:** 2022-07-19

**Authors:** Jerome Amir Singh, Dhayendre Moodley, Maggie Little, Florencia Luna, Katherine Littler, Nagalingeswaran Kumarasamy

**Affiliations:** ^1^ Howard College School of Law, University of KwaZulu‐Natal Durban South Africa; ^2^ Dalla Lana School of Public Health University of Toronto Toronto Ontario Canada; ^3^ Department of Obstetrics and Gynaecology, School of Clinical Medicine, and Centre for the AIDS Programme of Research in South Africa University of KwaZulu‐Natal Durban South Africa; ^4^ Kennedy Institute for Ethics and Department of Philosophy Georgetown University Washington DC USA; ^5^ CONICET (National Scientific and Technological Research Council) FLACSO Buenos Aires Argentina; ^6^ Global Health Ethics & Governance Unit, Research for Health Department World Health Organization Geneva Switzerland; ^7^ VHS Infectious Diseases Medical Centre Voluntary Health Services Chennai India

**Keywords:** ethics, exclusion, pregnant, breastfeeding, vulnerability, HIV/AIDS

Pregnant and breastfeeding women (PBW) need new antiretroviral (ARV) agents and drug delivery technologies that are safe and effective for treatment and prevention of HIV. Perhaps unsurprisingly, this group is underrepresented in studies investigating new preventive or therapeutic interventions, with foetal safety commonly cited as a reason to exclude pregnant women from research [1]. This exclusion has precipitated critical knowledge gaps, including in the field of HIV/AIDS, thereby delaying PBW's access to better ARV treatment or preventive agents and drug delivery technologies (e.g. administration through extended‐release/long‐acting injections, infusions or implants).

In 2020, an international, interdisciplinary and intersectoral working group convened as part of the PHASES (Pregnancy and HIV/AIDS: Seeking Equitable Study) Project, funded by the U.S. National Institutes of Health, identified critical evidence gaps in optimal dosing during pregnancy, foetal safety and maternal outcomes associated with new ARV agents and provided ethical guidance to end these evidence gaps [2]. Further guidance on how and when pregnant women should be included in research investigating new ARVs and drug delivery technologies emerged from a workshop convened by the World Health Organization (WHO) and the International Maternal Pediatric Adolescent AIDS Clinical Trials (IMPAACT) Network [3]. Building on these landmark documents, this work briefly reiterates the marginalization of PBW in studies investigating new ARVs, and further argues that PBW should be considered distinct populations for inclusion in studies of novel ARV agents.

Historic factors have contributed to PBW being excluded from studies investigating new ARV agents and drug delivery technologies [4], many of which still persist today. These include misunderstandings about regulatory mechanisms that govern research involving drugs in PBW, concerns about the legal liability of drug developers, sponsors and investigators, and a protectionist culture around such population participating in research [4]. This characterization has stereotyped PBW as a “vulnerable” population in need of protection [5]. For example, while research ethics guidance published by the Council for the International Organization of Medical Sciences (CIOMS) and the WHO (hereinafter the “CIOMS Guidelines”) notes: “Pregnant women must not be considered vulnerable simply because they are pregnant” [6], until recently, the United States Code of Federal Regulations (CFR) characterized pregnant women as “vulnerable” [7]. However, vulnerability is contextual, multiple‐layered and dynamic [8]. Some ethicists argue that using this designation for pregnant women is inappropriate and disrespectful [9].The CIOMS Guidelines notes: “A direct consequence of the routine exclusion of pregnant women from clinical trials is their use of medications (both prescription and non‐prescription) lacking data from clinical trials about the potential individual benefits and harms to themselves, their foetuses and their future children” [6]. In 2017, the CFR revised its language, dropping pregnant women from the list of vulnerable research populations [10].

Despite such guidance at the international level, PBW are still routinely excluded from studies investigating new ARV agents. Ironically, such a blanket exclusion heightens vulnerability and is counter to the health interests of PBW—and their infants. PBW living with HIV, like non‐pregnant women, need access to optimal HIV treatments, both to reduce vertical transmission of HIV and to safeguard their own health. In addition, PBW who are at high risk of HIV transmission and are having unprotected sex during pregnancy and postpartum, like their non‐pregnant and non‐breastfeeding counterparts, should have access to effective and safe options of pre‐exposure prophylaxis (PrEP). Many women engage in sexual activity during pregnancy and resume sexual activity early after childbirth without the use of contraception [11, 12]. WHO recommends daily PrEP for women who are at high risk of HIV transmission and having unprotected sex during pregnancy [13]. Despite WHO noting that biological factors increase a woman's susceptibility, and social and behavioural factors may increase exposure, to HIV infection, and moreover, that PBW who acquire HIV during this period have a greater risk of transmitting HIV to their infant than women who acquired HIV before pregnancy [13], PBW have been excluded from PrEP clinical trials. Moreover, for both treatment and prevention trials, women of reproductive age who are not pregnant are usually required to use dual contraception to participate in registrational trials [14, 15], while participants who become pregnant during a trial are often required to discontinue study drugs [16]. While such exclusion criteria and trial enrolment conditions are supposedly aimed at protecting the interests of foetuses and infants, an ethics‐centric approach dictates that HIV research should be responsive to the need to gather evidence on the full range of people who could benefit from the drug. The interests of PBW as a population are best served through their responsible enrolment in research, rather than their blanket exclusion. Epidemiological context also matters. For instance, the need to include PBW women in HIV clinical trials is arguably greater in settings with high HIV prevalence rates among women of reproductive age.

The criteria of responsible enrolment are clearly important. The inclusion of any population in drug research, especially interventional drug research, requires the assessment of the potential risks of such inclusion, as weighed against potential benefits in comparison with the risk/benefit profile of existing options. It also requires ensuring valid consent. In the context of ARV research with PBW, these criteria—spelled out in the above guidance and reflecting current regulatory and bioethical principles—include assessment of the potential risk and benefit to woman and her foetus, as compared to existing safe and effective ARV agents [2, 6].

While the field of ethics increasingly—and rightly—advocates for the inclusion of PBW in biomedical research, we also believe that the needs of PBW are best served if they are treated as distinct populations for purposes of ethics review and policy recommendations. Several ARVs are known to have altered pharmacokinetic profiles during pregnancy [17]. However, dosing concerns during pregnancy may not necessarily apply to breastfeeding women. Further, PBW are distinct with respect to their HIV‐related risks and challenges they face with HIV treatment [11], and consequently, the potential HIV‐related risks their offspring may face.

Including PBW in a trial involves careful risk–benefit assessments, both in relation to the mothers and their foetuses or offspring; but these risk–benefit assessments will often differ when considering the contexts of pregnancy versus lactation. For instance, in cases when pregnancy would be justified as an exclusion ground based on the potential teratogenic risks study drugs may pose to a foetus, or the lack of safe and effective dosing data in pregnancy, it may be appropriate for breastfeeding women to enrol in HIV treatment and prevention clinical trials, when there are reassuring data of low level of drug in breastmilk. Such data are available from animal models or studies where the drug was used for a different indication. For example, tenofovir diphosphate fumarate (TDF) is extensively studied in women living with HIV as part of an ARV combination treatment or hepatitis treatment in PBW, and such studies include breastmilk concentrations of TDF/FTC [18, 19].

A woman who becomes pregnant while enrolled in a clinical trial should be unblinded and allowed to continue on the investigational drug if the potential benefits of continued treatment for the woman outweigh the risks of ongoing foetal exposure to the investigational drug, of discontinuing maternal therapy and/or of exposing the foetus to additional drugs if placed on an alternative therapy [20]. Such participants should undergo a second informed consent process that reflects these additional risk–benefit considerations [20]. If a woman's trial participation is stopped upon pregnancy, she should be permitted to resume her participation, postpartum. While prenatal and postpartum care are often viewed as a part of a continuum, PBW should be treated as separate groups for purposes of research guidelines, with distinct behaviours, needs, physiologic considerations and risk profiles. For both populations, however, the current status quo of blanket exclusion on precautionary grounds is inequitable and unethical. Instead, PBW exclusion should be assessed based on the specific potential risk/benefit profile of a given trial.

Including PBW in a trial involves careful risk–benefit assessments, both in relation to women and their offspring. The inclusion of PBW in HIV clinical trials and their consideration as distinct cohorts will not just address critical knowledge gaps; doing so is in the interests of public health and should be considered an ethical imperative.

## COMPETING INTERESTS

None declared.

## AUTHORS’ CONTRIBUTIONS

JAS conceptualized and drafted the manuscript following input from DM, ML, FL, KL and NK. All authors contributed to successive iterations of the manuscript and approved the final iteration.

## FUNDING

JAS, DM, ML, KL and NK: None declared. FL: Research reported in this publication was supported by the Fogarty International Center of the National Institutes of Health under Award Number R25TW001605.

## DISCLAIMER

The content is solely the responsibility of the authors and does not necessarily represent the views of their employers or funders.
